# The first genetic map for yellow lupin enables genetic dissection of adaptation traits in an orphan grain legume crop

**DOI:** 10.1186/s12863-019-0767-3

**Published:** 2019-08-14

**Authors:** Muhammad Munir Iqbal, Mark Huynh, Joshua A. Udall, Andrzej Kilian, Kedar N. Adhikari, Jens D. Berger, William Erskine, Matthew N. Nelson

**Affiliations:** 1 School of Agriculture and Environment, The University of Western Australia, Perth, WA Australia; 2 Centre for Plant Genetics and Breeding and Institute of Agriculture, The University of Western Australia, Perth, WA Australia; 3 The College of Life Sciences, Brigham Young University, Provo, UT USA; 4 USDA-ARS Southern Plains Agricultural Research Center, 2881 F&B Rd., College Station, TX 77845 USA; 5 Diversity Arrays Technology, University of Canberra, Canberra, Australia; 6 School of Life and Environmental Sciences, I A Watson Grains Research Centre, The University of Sydney, Narrabri, NSW Australia; 7 Agriculture and Food, CSIRO, Floreat, WA Australia; 8 The UWA Institute of Agriculture, Perth, WA Australia

**Keywords:** Yellow lupin, Linkage map, Adaptation traits, Phenology, QTL analysis

## Abstract

**Background:**

Yellow lupin (*Lupinus luteus* L.) is a promising grain legume for productive and sustainable crop rotations. It has the advantages of high tolerance to soil acidity and excellent seed quality, but its current yield potential is poor, especially in low rainfall environments. Key adaptation traits such as phenology and enhanced stress tolerance are often complex and controlled by several genes. Genomic-enabled technologies may help to improve our basic understanding of these traits and to provide selective markers in breeding. However, in yellow lupin there are very limited genomic resources to support research and no published information is available on the genetic control of adaptation traits.

**Results:**

We aimed to address these deficiencies by developing the first linkage map for yellow lupin and conducting quantitative trait locus (QTL) analysis of yield under well-watered (WW) and water-deficit (WT) conditions. Two next-generation sequencing marker approaches - genotyping-by-sequencing (GBS) and Diversity Array Technology (DArT) sequencing - were employed to genotype a recombinant inbred line (RIL) population developed from a bi-parental cross between wild and domesticated parents. A total of 2,458 filtered single nucleotide polymorphism (SNP) and presence / absence variation (PAV) markers were used to develop a genetic map comprising 40 linkage groups, the first reported for this species. A number of significant QTLs controlling total biomass and 100-seed weight under two water (WW and WD) regimes were found on linkage groups YL-03, YL-09 and YL-26 that together explained 9 and 28% of total phenotypic variability. QTLs associated with length of the reproductive phase and time to flower were found on YL-01, YL-21, YL-35 and YL-40 that together explained a total of 12 and 44% of total phenotypic variation.

**Conclusion:**

These genomic resources and the QTL information offer significant potential for use in marker-assisted selection in yellow lupin.

**Electronic supplementary material:**

The online version of this article (10.1186/s12863-019-0767-3) contains supplementary material, which is available to authorized users.

## Background

Being sessile organisms, plants must adapt to the environments in which they find themselves. This is achieved primarily by genetic adaptation. Key adaptation traits such as abiotic stress tolerance, are typically complex and controlled by several genes. Quantitative trait locus (QTL) analysis is a powerful tool to investigate the genetic control of complex traits and can be used to identify linked molecular markers for use in marker-assisted selection (MAS) [1–3]. QTLs are identified by integrating phenotypic measurements with genome-wide marker information either in purpose-made experimental populations (conventional linkage QTL analysis) or in a diverse panel of unrelated lines (association QTL analysis) [4]. A pre-requisite of QTL analysis is the availability of a genetic map or genome sequence in which regions of the genome controlling quantitative traits can be delineated.

Genetic maps can be made using molecular markers and segregating populations. A wide range of marker systems have been developed and applied in legumes to generate linkage maps and aid genome assembly [5, 6]. Such marker systems have been employed for QTL mapping for adaptation and yield traits in a range of legume species including chickpea (*Cicer arietinum* L.), lotus (*Lotus japonicus* L.)*,* barrel medic *(Medicago truncatula* Gaertn.), faba bean (*Vicia faba* L.), field pea (*Pisum sativum* L.), red clover (*Trifolium pretense L.*), peanut (*Arachis hypogaea* L.), common bean (*Phaseolus vulgaris* L.) and white clover (*Trifolium repens* L.). As a result, several significant QTLs controlling these traits were identified for further use in MAS [7–16].

Yellow lupin (*Lupinus luteus* L., 2n = 52) is an annual grain legume which offers advantages over its sister domesticates: narrow-leafed lupin (*L. angustifolius* L.) and white lupin (*L. albus* L.). Yellow lupin is adapted to acid soils [17], is more water-logging tolerant [18] and has enhanced resistance against cucumber mosaic virus [19]. It has the highest protein content (average of 45%) of domesticated lupins and an oil content of 6% making it a candidate for human food and aquaculture feed, as well as animal feed [20–22].

Despite this promise, yellow lupin has not been generally embraced by farmers because of its low productivity compared to narrow-leafed lupin. Consequently, more focus has been given to the narrow-leafed lupin on research [22]. Apart from some studies on yellow lupin domestication traits and disease tolerance potential [23–25], we lack information on yellow lupin adaptation, its physiology in diverse environments and the genetics controlling these adaptation traits. This lack of knowledge prompted the current study.

A serious impediment to making progress in yellow lupin adaptation and breeding is the limited knowledge available on genomic resources with mere two RNAseq datasets [26, 27]. No linkage map or reference genome has been reported. In contrast, these resources are available to its close relatives narrow-leafed lupin and white lupin, which have allowed investigation of genomic regions controlling yield, nutritional, domestication and physiological traits on these species [27–38]. Identification of genomic regions controlling desirable traits (high yielding, low alkaloid, indehiscence and adaptation to diverse environments) would help researchers efficiently select for those traits through MAS in order to adjust these adaptive traits for the development of more sustainable and resilient yellow lupin production [39–42].

With the rapid advances in next generation sequencing (NGS) technologies, the cost of genomic analysis has fallen significantly. Entire mapping populations can be genotyped resulting in the generation of millions of genomic data points and thousands of markers [11, 43, 44]. These approaches could be used to improve our understanding of the yellow lupin genome and to enable QTL analysis of adaptation and phenology traits. In this study, we report the first yellow lupin linkage map and use it to conduct a QTL analysis of plant productivity and phenology under well-watered (WW) and water-deficit (WD) conditions.

## Results

### Marker discovery

Using the GBS approach, a total of 13,462 SNP markers were discovered. Preliminary mapping using relaxed filtering (< 25% missing values and < 25% heterozygosity) led to illegitimate fusion of linkage groups (data not presented). Therefore, increased stringency was applied After filtering markers based on quality parameters (< 6.4% heterozygous values and < 10% missing values), which left 948 high quality SNP markers (prefixed ‘SCAFFOLD’). We considered these insufficient to develop a new and comprehensive linkage map. Additional markers were developed using the DArT-seq approach. Two categories of DArT-seq markers were discovered: 5,590 SNP and 8,854 PAV (presence/absence variation) markers. After quality filtering these markers based on the above threshold, a total of 1,049 SNP (prefixed ‘DArT-SNP’) and 957 PAV (prefixed ‘DArT-PAV’) markers were retained, giving a total of 2,945 markers in 97 RILs for linkage map development.

### Linkage map development

Linkage mapping was performed with the aid of MultiPoint 3.3 using 2,945 markers. Linkage groups containing 5 or more markers were considered as the framework genetic map. The framework genetic map consisted of 40 linkage groups representing yellow lupin’s 26 chromosomes. These linkage groups contained a total of 919 framework markers along with 1,262 redundant markers (exactly similar to framework markers but with more missing values). A total of 277 (majority of them were DArT markers), potentially problematic markers, were moved to the MultiPoint section termed the ‘Heap’, either due to moderately high segregation distortion (Chi-square 0.0001 < *P* < 0.001) or due to disturbances in the monotonic increase in recombination frequencies along linkage groups, which is normally caused by genotyping errors [45]. These markers were subsequently attached to most likely interval in the established framework map, thus comprising of 2,458 markers in total. A total of 487 out of 2,945 markers mapped to small linkage groups of less than five framework markers or remained unlinked singletons and were excluded from the final map.

**Fig. 1:**
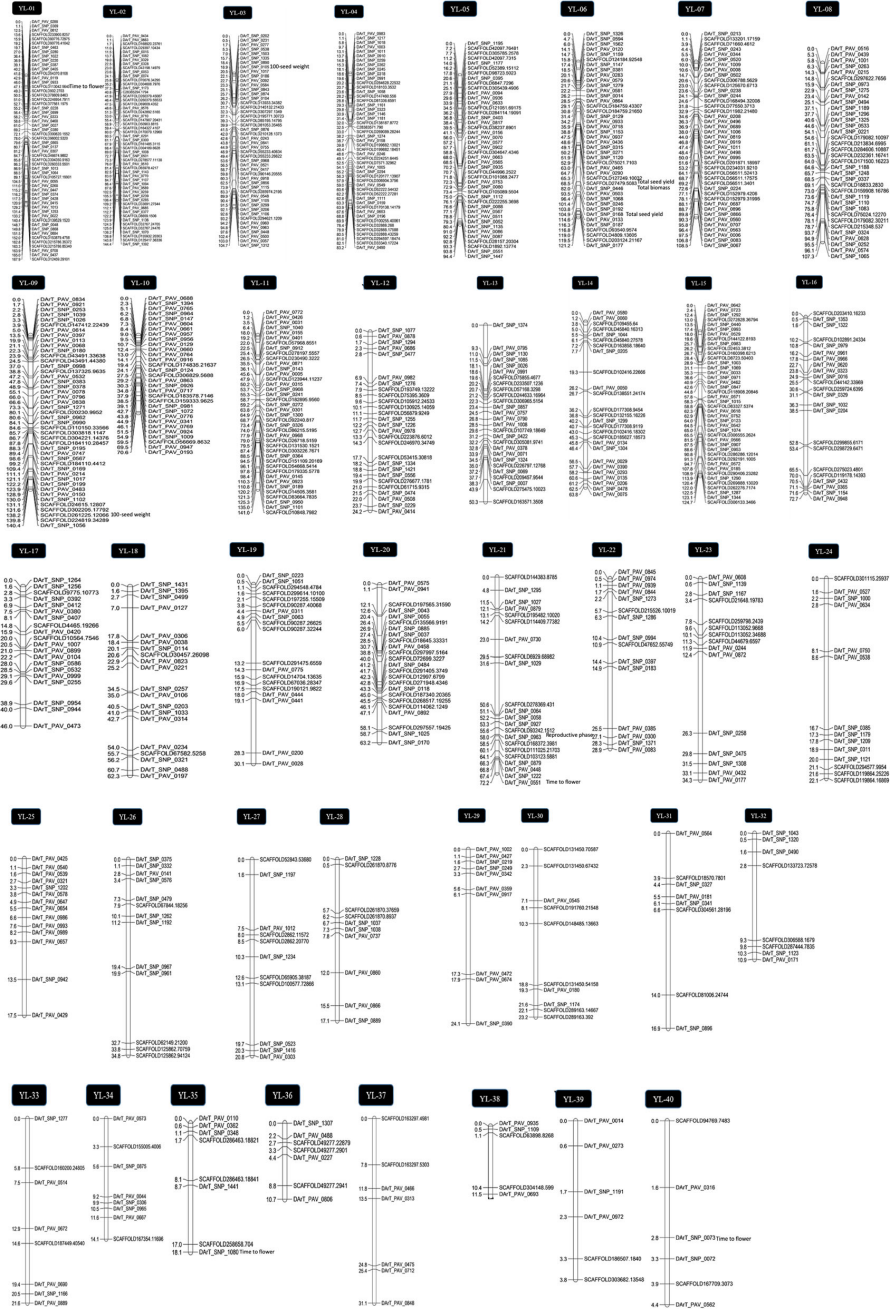
A genetic map of yellow lupin based on 912 framework markers in 40 linkage groups (YL01-YL40). Each vertical bar represents a linkage group with marker names on right side of the bar while the position in Kosambi centiMorgan are on the left side of the bar. The markers prefixed ‘SCAFFOLD’ represents SNP markers from genotyping-by-sequencing while markers prefixed ‘DArT-SNP’ are SNP markers from DArT-seq and ‘DArT-PAV’ are presence/absence variant markers from DArT-seq. Full marker data including redundant and attached markers are provided in Additional file 2: Table S1

The length of linkage groups ranged from 3.8 to 167.9 cM with an average of 56.5 cM, while the average interval size among loci on each linkage group ranged from 0.76 cM to 5.18 cM with an average size of 2.29 cM (Table 1, Fig. 1). The maximum interval size was 12.8 cM. The length of the entire linkage map was 2,261.3 cM.

**Table 1:** Summary of yellow lupin linkage groups (YL-01 to YL-40) comprising numbers of framework markers, redundant markers, attached markers, total no. of loci, linkage group length and average interval size

Linkage Group	Framework markers	Redundant markers	Attached markers	Total # loci	Length of linkage group (cM)	Average interval size (cM)
YL-01	56	46	1	103	167.9	3.00
YL-02	53	60	65	179	144.4	2.72
YL-03	48	87	47	182	104.7	2.18
YL-04	48	106	22	177	83.2	1.73
YL-05	44	53	19	116	94.4	2.15
YL-06	42	23	7	72	121.2	2.89
YL-07	42	16	0	58	108.5	2.58
YL-08	36	45	9	90	107.3	2.98
YL-09	45	87	0	132	140.4	3.12
YL-10	31	64	3	98	70.6	2.28
YL-11	40	63	0	103	141	3.53
YL-12	27	57	47	131	24.2	0.90
YL-13	27	110	5	142	50.3	1.86
YL-14	26	9	1	36	63.8	2.45
YL-15	42	102	5	149	124.7	2.97
YL-16	23	15	1	39	72.7	3.16
YL-17	20	29	6	55	46	2.30
YL-18	20	42	6	68	62.3	3.12
YL-19	19	13	3	35	30.1	1.58
YL-20	24	55	14	93	63.2	2.63
YL-21	22	21	1	44	72.2	3.28
YL-22	15	10	3	28	28.9	1.93
YL-23	15	7	6	28	34.3	2.29
YL-24	14	13	0	27	22.1	1.58
YL-25	14	38	1	53	17.5	1.25
YL-26	13	9	4	26	34.8	2.68
YL-27	11	8	0	19	20.8	1.89
YL-28	10	2	0	12	17.1	1.71
YL-29	10	4	0	14	24.1	2.41
YL-30	10	18	0	28	23.2	2.32
YL-31	8	11	0	19	16.9	2.11
YL-32	8	6	0	14	10.9	1.36
YL-33	8	4	0	12	21.6	2.70
YL-34	8	11	0	19	14.1	1.76
YL-35	8	2	0	10	18.1	2.26
YL-36	7	4	1	12	10.7	1.53
YL-37	7	3	0	10	31.1	4.44
YL-38	5	1	0	6	13.8	2.76
YL-39	6	3	0	9	3.8	0.63
YL-40	6	4	0	10	4.4	0.73
Total/average	919	1262	277	2458	56.5	2.29

### ANOVA and QTL analysis of adaptation and phenology traits

The ANOVA revealed that moisture treatment, genotypic and interaction effects were significant (*P* < 0.001) for total seed yield, total biomass, 100-seed weight, time to flower and length of reproductive phase (Table 2). Among the main effects, water regime differences were far more influential than genotypic effects for all traits except time to flower (Table 2). Small but significant interaction effects were observed for all traits, especially for total seed, where the interaction effect was statistically different (larger) than the genotypic effect (F value of 1.50 vs 1.55) (Table 2). The wild parent P28213 out yielded the domesticated parent Wodjil in both Total seed yield and Total biomass. The total seed-yield of P28213 was 204 g/m^2^ and 139 g/m^2^ under well-watered and water-deficit treatments respectively, while Wodjil exhibited the total seed yield of 134.5 g/m^2^ and 85 g/m^2^ under well-watered and water-deficit treatments respectively. Similarly, the total biomass of P28213 was 713 g/m^2^ and 629 g/m^2^ under well-watered and water-deficit treatments respectively, while Wodjil exhibited the total biomass of 477 g/m^2^ and 365 g/m^2^ under well-watered and water-deficit treatments respectively (Table 3). The 100-seed weight among both the wild and domesticated parents was not significantly different, As the parent P28213 showed the 100-seed weight of 10.6 g and 9.4 g under well-watered and water-deficit treatments respectively, while the 100-seed weight of Wodjil was 9.2 g and 10 g under well-watered and water-deficit treatments respectively (Tables 2 and 3). Average total seed yield of RIL population was 221 g/m^2^ under well-watered (WW) conditions whereas under water-deficit (WD), the yield was 114 g/m^2^. Most of this difference is attributable to difference in lateral stem productivity. The difference in main stem yield between moisture treatments was comparatively low with 132 g/m^2^ in WW and 99 g/m^2^ in WD treatment. By contrast, major differences were seen for lateral stem yield with a mean of 87 g/m^2^ recorded in WW and only 12 g/m^2^ in the WD treatment. The variances for total biomass among treatments were also found significant according to ANOVA and they measured 705 g/m^2^ and 480 g/m^2^ in WW and WD treatments respectively (Table 3).

**Table 2:** Split-plot ANOVA for 1) yield traits; total seed yield (g/m^2^), total biomass (g/m^2^) and 100-seed weight (g) and 2) Phenology traits; time to flower (days) and length of reproductive phase (days) in the yellow lupin RIL population under two moisture treatments i.e. well-watered and water-deficit. Moisture treatments were main blocks while genotypes were sub-plots in this experiment. Where d.f. is degree of freedom and MS is means sum of squares

	Total seed yield			Total biomass			100-seed weight			Time to flower			Length of reproductive phase		
Source	d.f. (m.v.)	MS	F	d.f. (m.v.)	MS	F	d.f. (m.v.)	MS	F	d.f. (m.v.)	MS	F	d.f. (m.v.)	MS	F
Treatment	1	55199.9	<.001	1	246994.9	<.001	1	319.1	0.022	1	416.6	0.245	1	38494.8	<.001
Error (a)	6	1446.1		6	5658.5		6	33.7		6	251.3		6	168.4	
Genotype	155	295.6	<.001	155	2192.0	<.001	155	16.5	<.001	155	73.7	<.001	155	78.9	<.001
Treatment x Genotype	154 (1)	206.7	<.001	154 (1)	1344.7	<.001	154 (1)	6.0	0.003	155	6.3	<.001	154 (1)	13.3	<.001
Error (b)	748 (182)	95.9		782 (148)	644.9		729 (201)	4.3		914 (16)	3.4		795 (135)	6.5	
Total	1064 (183)			1098 (149)			1045 (202)			1231 (16)			1111 (136)

**Table 3:** Means of total seed yield (g/m^2^), main stem seed yield (g/m^2^), lateral stem seed yield (g/m^2^), total biomass (g/m^2^), 100-seed weight (g) and time to maturity (d) in yellow lupin under well-watered and water-deficit treatments and their Least Significant Difference (LSD)

	RILs		Wodjil		P28213			
Treatments	Well-watered	Water-deficit	Well-watered	Water-deficit	Well-watered	Water-deficit	Overall mean	LSD
Total yield (g/m^2^)	221.0	114.3	134.5	85.0	204.0	139.0	168.0	14.2
Main stem seed yield (g/m^2^)	132.0	99.3	101.0	80.4	137.0	97.5	116.0	8.4
Lateral stem seed yield (g/m^2^)	87.0	12.0	48.0	0.0	67.0	23.0	49.5	6.4
Total biomass (g/m^2^)	705.0	480.0	477.0	365.0	713.3	629.0	592.5	36.1
100-seed weight (g)	11.3	10.2	9.2	10.0	10.6	9.4	10.75	2.9
Time to maturity (d)	140.0	130.0	140.0	130.0	140.0	130.0	135.0	2.9

As with the yield traits, water regime also showed a significant effect on the duration of reproductive growth although the moisture stress treatment was applied only after flowering. The differences for time to flower between treatments were non-significant as expected. Within moisture treatments genotypes differed significantly (*P* < 0.001) in time to flower, however difference among genotypes in maturity days were non-significant. Overall, the flowering duration and reproductive phase were reduced (*P* < 0.001) under WD treatment. This phenology difference resulted in reduction of time to maturity under WD environment. The time to flower from transplanting ranged between 70 and 87 days in both moisture treatments with the parent Wodjil flowering after 70 days and P28213 flowering 14 days later (Tables 2 and 3).

The regions of the yellow lupin genome contributing to these heritable traits were identified through QTL analysis. The small but significant interaction effect of water regime on total seed yield that was observed by ANOVA was supported by QTL analysis, which exclusively found only interaction QTLs (i.e. no main effect QTLs) for total seed yield (Tables 2 and 4). In contrast, the genotype main effect was relatively strong for total aerial biomass, 100-seed weight and phenology. In other words, the same QTLs contributed to the genotype main effect and were identified in both water regimes for these traits. Two significant QTLs (−log (10) > 3.82) on linkage groups YL-06 and YL-26 were found to be associated with total biomass. But the biomass QTL on YL-06 showed significant QTL x e interaction meaning that it was only detected in one environment. The QTL associated with biomass on linkage group YL-26 explained 9% of total phenotypic variation (Table 4).

**Table 4:** Summary of significant (*P* < 0.001) quantitative trait locus positions associated with different adaptation (total biomass, 100-seed weight, time to flower and length of reproductive phase) traits in yellow lupin, their heritability estimates, nearest marker to the QTL, position on map, their statistical significance (−log (10) values) and percent explained variance. QTL x E interaction at *P* < 0.001

Trait	Heritability (%)	Nearest marker	Linkage group (position in cM)	Percent explained variance (%)	-log (10) value	QTL x E
Total seed yield (g/m^2^)	10	SCAFFOLD27479.5083	Yl-06 (68.5)	10	7.52	yes (only in WW environment)
		DArT_SNP_0168	YL-06 (104.9)	5	5.76	yes (only in WW environment)
Total biomass (g/m^2^)	14	SCAFFOLD62149.21200	YL-26 (32.7)	9	3.88	no
		DArT_SNP_1056	Yl-06 (82)	27	10.18	yes (only in WW environment)
100-seed weight (g)	23	DArT_SNP_0105	YL-03 (18.9)	16	4.80	no
		SCAFFOLD261225.12066	YL-09 (138.2)	12	3.92	no
Time to flower (d)	71	SCAFFOLD113042.5640	YL-01 (47.1)	8	4.67	no
		DArT_PAV-0551	YL-21 (72.2)	15	5.91	no
		DArT_SNP_1080	YL-35 (18.1)	8	4.25	no
		DArT_SNP_0073	YL-40 (2.8)	12	4.82	no
Length of reproductive phase (d)	55	SCAFFOLD93242.1512	YL-21 (55.6)	12	4.56	no

Two main effect QTLs (−log (10) > 3.82) were found to be associated with 100-seed weight under both WD and WW environments. The two QTLs were located on linkage groups YL-03 and YL-09, which together explained 28.4% of total phenotypic variation in the population (Table 4).

Four significant (−log (10) > 3.82) loci were found to be associated with time to flower. These QTLs were found on linkage groups YL-01, YL-21, YL-35 and YL-40, which together explained 43% of total phenotypic variation in the population (Table 4).

Only one significant (−log (10) > 3.82) QTL was identified to be associated with reproductive growth period under both treatments. This QTL was found on linkage group YL-21 and explained 12% of total phenotypic variance (Table 4). Heritability (H^2^) estimates were high for phenological traits: time to flower (71%); length of the reproductive phase (55%), while they were low for yield traits: total seed yield (10%), biomass (14%), and 100-seed weight (23%) (Table 4).

## Discussion

Here we present the first reported linkage map of yellow lupin using GBS and DArT-seq methods to genotype a new wild x domestic F_8_ RIL population. The linkage map composed of 2,458 markers in 40 linkage groups. Furthermore, QTL analysis revealed significant loci controlling yield-related and phenology traits: total biomass, 100-seed weight, time to flower and length of reproductive phase.

### Linkage map development

We genotyped the RIL population along with parents using a genotyping-by-sequencing approach described by Poland et al., [46]. As a result, only 948 good quality SNPs markers (with < 10% missing values and low segregation distortion) out of a total of 13,462 originally discovered SNPs were selected for mapping. A large number of missing values are a common feature of GBS which could cause ambiguity in the true location of markers. While imputation methods appear to be efficient in using data matrices with a high proportion of missing values [47], linkage mapping is very sensitive to the systematic errors given the varying accuracy of imputation reads [48]. In a recent study, only 6% of sequence reads were found to be useful for genotype calls after filtering for genotype quality and missing data [49]. Therefore, we took a conservative approach of removing markers with excessive missing values.

The combination of restriction enzymes that produces highly polymorphic fragments is also critical for an efficient GBS protocol, and each organism may differ in the optimum set of restriction enzymes [50, 51]. Possibly a higher proportion of robust, polymorphic markers may have been achieved with a different combination of restriction enzymes (here, *Pst*I and *Taq*αI were used). This highlights the advisability of carrying out preliminary testing of different restriction enzyme combinations prior to embarking on large-scale genotyping of populations. Given that, the second NGS approach - DArT-seq – yielded a much higher number of robust, polymorphic markers using a different restriction enzyme pair than in our GBS approach: *Pst*I and *Mse*I. In total 5,590 SNP and 8,854 PAV markers were discovered and out of that a total of 1,049 SNPs and 957 PAV markers were retained for linkage mapping after filtering for missing values and genotype quality. The length of the entire linkage map was 2,261.3 cM, comparing well with the well-established maps of white lupin and narrow-leafed lupin genomes, where markers were distributed over 25 chromosomes and covered the 1,916 cM of the white lupin genome [28]. While narrow-leafed lupin map was comprised of 20 linkage groups covering a total of 2,361.8 cM of its genome [52].

The combination of GBS and DArT-seq methods greatly improved the number of markers with acceptable quality. A substantial number (2,945 out of original 27,906) of high-quality markers with low segregation distortion and very few missing values were achieved to develop the linkage map. The resultant SNP and PAV markers from both techniques integrated well. This huge reduction in the number of markers was in large part related to high segregation distortion from the expected 1:1 ratio. We removed markers deviating (*P* < 0.0001) from an expected 1:1 ratio of an F_8_ RIL population to avoid segregation distortion that may have illegitimately joined linkage groups containing similarly skewed markers, with subsequent effects on QTL analysis [53, 54]. At the same time, we were careful not to apply an excessive filtering that would remove genuinely skewed regions of the genome, such as those containing domestication traits (for example, the low-alkaloid *iucundus* genes in narrow-leafed lupin [52, 55]. The markers associated with low alkaloid in yellow lupin was also highly segregated but was captured the current levels of stringency (data not presented; manuscript in preparation). The wide crosses with wild material often used for RIL population development for linkage mapping due to the typically high level of marker polymorphism [56], However, reduced recombination in such crosses can also cause high segregation distortion [57]. Given that, we conclude that this phenomenon may have occurred in our experimental RIL population as it was developed by employing a wide cross. The use of cluster analysis was novel in terms of identifying segregation distortion in this population so that the subsequent analysis could be conducted on an unbiased subset.

In total, we identified 40 linkage groups to represent the 26 chromosome pairs of yellow lupin. This excess number of linkage groups highlights that this remains a first draft linkage map. Development of further genomic resources and suitable mapping populations of yellow lupin would facilitate the refinement of this map and the production of more complete map with the 26 linkage groups expected for this species.

### QTL mapping

The new genetic linkage map was used as the basis for the first QTL analysis of yellow lupin, which identified genomic regions controlling total biomass, 100-seed weight and phenology under different moisture conditions. The effect of QTLs associated with biomass and 100-seed weight was 14 and 23% respectively, while the effect of QTLs associated with time to flower and length of reproductive phase was 71 and 55% respectively (Table 4). This compares favourably to similar studies conducted in other legumes where QTLs associated with adaptation traits captured 5–69% of phenotypic variation [4, 7, 8, 10, 12]. It was notable that the main effect QTLs associated with important yield traits such as total biomass, 100-seed weight, time to flower and length of reproductive phase were not significantly affected by the water treatments applied in this study. This is positive news for lupin breeders as it suggests that superior alleles can be selected in a range of water regimes. However, total seed yield, which is one of the most important traits could only be detected under WW conditions, suggesting that it may prove difficult to identify QTLs for improved yield under WD conditions. However, the small plot size in our studies enforced by limited space under a rainout shelter inflated the residual error that reduced both heritability and our capacity to detect significant QTLs.

The yield QTLs were not associated with phenology, so it should be possible to introduce these higher yield alleles without compromising early phenology, a drought avoidance trait which is essential for reliable yields in Australian growing regions that experience severe terminal drought. These results highlight that the yellow lupin breeding effort is still in its infancy, and greatly improved yields could be achieved if given the opportunity to conduct further rounds of crossing and selection. Currently there is no active yellow lupin breeding in Australia. If breeding programs were to be established, NGS-derived SNP markers developed here can readily be converted to single locus assays for use in marker-assisted selection to aid any renewed breeding effort [58, 59].

From an ecophysiological perspective, the lack of interaction between moisture regime and QTLs for yield traits (total biomass and seed weight) and phenology was unexpected, given that water deficit is likely to select for drought escape, whereas longer season, higher-rainfall environments may favour delayed phenology to maximize biomass [60]. This may be because maturity differences between WW and WD environments were relatively small i.e. 10 days probably because of forced maturity at the end of the season? (Table 3). We expect that if the same experiment was conducted in a longer season environment with greater contrast between WW and WD treatments, we would have detected a stronger role of phenology on productivity.

The QTLs associated with time to flower explained a modest amount of 44% of total phenotypic variation in a trait with 71% heritability. On the other hand, QTLs controlling very low heritable yield traits only explained 9–28% of total phenotypic variation. The results suggest the presence of valuable diversity in the experimental germplasm that could be utilised for crop improvement.

## Methods

### Recombinant-inbred line (RIL) population development

An experimental recombinant-inbred line (RIL) population was developed from a wide bi-parental cross between a wild yellow lupin accession P28213 and an Australian cultivar Wodjil (a selection from Polish cultivar Teo) at the Department of Primary Industries and Rural development (South Perth, Western Australia). The parents were selected on the basis of contrast in adaptation traits such as phenology, below/above biomass, and response to terminal water stress and domestication traits [60]. Wodjil was bred for short season environments and exhibits ruderal traits such as early phenology, low above/below ground biomass and low yield potential and exhibits a drought escape strategy. The wild parent P28213 originates from a high-moisture environment (average seasonal rainfall 1163 mm) in the Azores (38.70 N, − 27.22 W) and exhibits competitive traits such as delayed phenology, high above/below biomass and high yield potential, but is prone to early water stress onset. Parents also differ in key domestication traits such as vernalisation response, growth habit, seed dehiscence, alkaloid content, seed permeability, and both flower and seed coat colour. The experimental RIL population was developed from a single F_1_ plant. One F_1_ individual was grown in a screen house and 300 F_2_ seeds were harvested. All F_2_ seeds were scarified by hand (due to hard seed coat segregating in the population) and sown in a screen house to obtain F_3_ seeds. Single seeds were taken from individual F_3_ plants and progressed to the F_8_ generation by single seed descent [61] to produce a total of 202 recombinant inbred lines. This RIL population of 202 lines was multiplied prior to phenotyping in a screen house and a total of 156 RILs were randomly selected for phenotyping and genotyping.

### Genotyping of RIL population

Genotyping of the RIL population was conducted using two NGS approaches: Genotyping-by-sequencing and DArT-seq methods.

### Genotyping-by-sequencing (GBS)

DNA extraction from 156 RILs and parents was performed using Qiagen DNeasy Plant 96 kit and Quant-iT™ PicoGreen (Life Technologies, Carlsbad, California) for DNA quantification. DNA for each genotype was normalised to the concentration of 40 ng/ul. Libraries were prepared for GBS using the protocol of Poland et al. [46]. Briefly, *Pst*I-HF and *Taq*αI restriction enzymes were used to digest DNA samples. A total of 96 barcoded adapters [46] for downstream identification were ligated to the 5′ end of digested DNA fragments, while a Y-shaped adapter was ligated to the 3′ end. PCR was used to amplify the resultant fragments along with the addition of Illumina adapters. The PCR-product was cleaned by using a Promega SV Wizard Gel Clean-Up System (Promega Corporation, Madison, Wisconsin). Samples were sent for Illumina HiSeq analysis by Beijing Genome Institute (BGI) at University of Davis, California for 150 bp paired-end sequencing.

GBS reads were trimmed based on quality parameters using Sickle [62] and were subsequently demultiplexed using a custom Perl script. Every pair of reads was isolated based on its exact match with one of the barcodes, which were then trimmed. The GSNAP program [63] was employed to map the reads to an unpublished SOAPdenovo genome assembly of *L. luteus* line ‘9242X4’, which had been produced using short-read denovo assembler developed by Luo et al., [64] (Joshua Udall, unpublished data). SAMtools [65] was used to produce BAM alignment files.

BamBam tools [66] were employed to process BAM files including single nucleotide polymorphism (SNP) calling, imputation and characterization. SNP markers were accepted if they had a minimum of 3 read coverage, had < 30% missing genotypes and minor allele frequency of ≥0.1. Imputation of missing genotypes was carried out by K-Nearest neighbour with k = 10. Further quality control was performed during linkage mapping. The number of markers (948 SNPs) obtained from this approach were not considered enough to create linkage map, hence, another genotyping method-DArT-seq was employed to obtain additional good quality markers.

### DArT-Seq

DNA isolation was performed on 156 RILs and parents using the CTAB method [67]. Qubit fluorimetry (Invitrogen, Carlsbad, CA, USA) was used for DNA quantification and results were confirmed by tallying with the corresponding band brightness on gel electrophoresis wells. DNA was normalised at the concentration of 50 ng/ul. Samples were sent for library preparation (using restriction enzymes *Pst*I and *Mse*I) and sequencing to Diversity Arrays Technology Pty Ltd. (Canberra, Australia).

Trimming of DArT-seq reads involved removal of the reverse adapter only and was not based on Illumina quality parameters but rather by alignment of multiple sequences and the consensus was taken across the population. The minimum average read depth of 2 for the reference allele and 1.5 for the alternative allele was used. For PAV (presence /absence variant) markers the minimum average allele read depth was 5.

### Linkage map development

After SNP calling from both GBS and DArT-seq pipelines, output loci were subjected to additional filtering. Those loci which showed significant (*P* < 0.0001) segregation distortion from the Mendelian expectation of 1:1 parental alleles were excluded from the analysis. Marker x RIL combinations with > 10% missing values were removed, thus leaving a total of 140 RILs out of 156 RILs for linkage map development. Initial mapping with 140 RILs in MultiPoint 3.3 software [68] failed to produce satisfactory linkage groups, which led us to investigate the structure of the RIL population. The NTSYS program [69] was employed to generate distance matrixes among RILs, which were then visualised by cluster analysis (Additional file 1: Figure S1) in Primer6 software [70]. Rather than the random genetic relationships expected within a RIL population that had been developed by single seed descent, there was distinct clustering of 43 RILs with the domesticated parent ‘Wodjil’, possibly indicative of unintended cross-pollination with domesticated-types of yellow lupin during single seed descent or seed admixture. Therefore, these 43 RILs were excluded from further linkage mapping to minimize bias. Thus, 97 F_8_ RILs were used for the final linkage map development in MultiPoint3.3. All loci with Chi^2^ *P* < 0.0001 and missing values > 10 were removed at the beginning of linkage analysis. Moderately distorted loci (*P* < 0.001) were moved to the ‘Heap’ within the MultiPoint linkage analysis but were not used to calculate linkage groups since segregation distortion may have led to illegitimate joining of separate linkage groups. Instead, such markers were allocated approximate genetic positions as ‘attached’ markers at the end of the analysis. Initial clustering was started at recombination fraction (rf) of 0.05. Marker ordering in each linkage group was performed in Multipoint and jack-knife re-sampling enhanced the robustness of marker order by keeping only markers with jack-knife value of > 90%. Those markers were designated as ‘framework’ markers. Other markers which mapped to the same location as framework markers were termed ‘redundant markers’ and they were assigned the same genomic location on the map as the framework markers. The same procedures were followed at each clustering cycle gradually increasing from recombination frequencies from 0.05 to 0.24. Manual inspection of clusters at each step helped to distinguish valid cluster mergers (two progenitor clusters most closely linked through their terminal loci) from invalid clusters (two progenitor clusters most closely linked through non-terminal markers). Joining of valid clusters was accepted, while invalid joining of clusters was rejected. Typically, the markers causing spurious linkage between clusters had higher segregation distortion values and/or missing values.

Upon the completion of the framework map, interval size values were transformed to account for multiple generations involved in F_8_ RIL population development and expressed in Kosambi centiMorgans (cM). Linkage groups were drawn using MapChart 2.3 [71].

### Phenotyping of RIL population

#### Experimental procedures and design

This experiment was conducted in a split-plot design in 2013 in a rain-out shelter at CSIRO Floreat (31°56′53.5″S 115°47′52.4″E), WA, Australia [72]. Where water regimes were placed as main plots and genotypes as sub-plots. A total of 156 recombinant inbred lines with sufficient number of seeds along with both parents Wodjil and P28213 were studied for their response to limited moisture and WW conditions. Experimental seed was scarified to remove the effect of variation in hard-seededness in the population. Imbibed seeds were vernalized by growing in Jiffy pots (Garden City Plastics Pty Ltd) at 8 °C for 3 weeks from 16th May, 2013 to avoid the confounding effect of variation in vernalization response in the experiment. All plant material was transplanted into the field on 10th June, 2013. Rhizobium inoculation was undertaken at transplantation into the field to promote nodulation. Manual weeding was done as needed.

Genotypes were grown under two water regimes applied to contiguous regions of a single field: a) WW: this treatment was kept watered from sowing till ripening and b) WD: terminal drought administered using an automatic rainout shelter after the onset of pod set. There were four replications of genotypes, with the two parents (Wodjil and P28213) replicated 8 times within each treatment. DiGGer package of R software was employed for spatially optimized randomisation [73]. A rainout shelter of dimension 11 × 14.5 m was used for the WD treatment with a frame area 0.5 m wide retained empty to reduce border effects. An immediately adjacent field area of similar dimensions was used for WW treatment. Sub-plot size was 0.25 × 0.5 m. Each plot was planted with five seeds 10 cm apart within a row and the row-to-row distance was 25 cm.

#### Moisture treatment

The WD treatment was applied at the post-anthesis stage when the first pod had developed on the main stem, while the plots of late flowering lines under the rainout shelter were individually irrigated until first podding. All the plant material grown under WW treatment was maintained with irrigation (rain or reticulation if required) in the open field.

From the time of the application of the WD treatment, soil moisture was measured 0, 15, 30 and 45 days post-stress imposition at three depths (0–20, 20–40 and 40–60 cm). The maximum, minimum and average temperature data and rainfall were obtained from Bureau of Meteorology, Australia website www.bom.gov.au for metrological station Shenton Park, WA 31.94°S, 115.79°E (approximately 2.9 km from the experimental site).

#### Trait measurements

Measurements were made for total seed yield, seed yield from main and lateral stems (in g/m^2^), total biomass (dry weight of aerial plant mass in g/m^2^), 100-seed weight (g), time to flower (days) and length of reproductive phase (days). The time to flower was measured from the day of transplanting until 50% of plants in a plot flowered while the length of reproductive phase was calculated by subtracting the time to flower from time to maturity.

#### Analyses of phenotypic data and quantitative-trait loci (QTLs) for adaptation traits

ANOVA was used to analyze quantitative traits in a split-plot model with water regime in main plots and genotype as sub-plots in GenStat version 17 (VSN International, UK). Residual plots were generated to visualize ANOVA assumptions and identify outliers. Heritability (H^2^) for traits studied separately in both treatments was calculated by the following formula:

$$ \mathrm{H}\mathbf{2}={\boldsymbol{\sigma}}^{\mathbf{2}}\boldsymbol{g}\raisebox{1ex}{$\left(\boldsymbol{ms}\left(\boldsymbol{g}\right)-\frac{\boldsymbol{ms}\left(\boldsymbol{e}\right)}{\boldsymbol{r}}\right)$}\!\left/ \!\raisebox{-1ex}{${\boldsymbol{\sigma}}^{\mathbf{2}}\boldsymbol{p}\ \left({\boldsymbol{\sigma}}^{\mathbf{2}}\boldsymbol{g}+{\boldsymbol{\sigma}}^{\mathbf{2}}\boldsymbol{e}.\right)$}\right. $$

where r is the number of replications, (*σ*) is variance components, ms is mean square values of genotype (g), phenotype (p) and error (e) [74]. Heritability of those traits which were compared among both treatments was calculated by the following formula [75]:

$$ \mathrm{H}\mathbf{2}={\boldsymbol{\sigma}}^{\mathbf{2}}\boldsymbol{g}\raisebox{1ex}{$\left(\boldsymbol{ms}\left(\boldsymbol{g}\right)-\frac{\boldsymbol{ms}\left(\boldsymbol{e}\right)}{\boldsymbol{r}\ast \boldsymbol{t}}\right)$}\!\left/ \!\raisebox{-1ex}{${\boldsymbol{\sigma}}^{\mathbf{2}}\boldsymbol{p}\ \left({\boldsymbol{\sigma}}^{\mathbf{2}}\boldsymbol{g}+{\boldsymbol{\sigma}}^{\mathbf{2}}\boldsymbol{e}\right)$}\right. $$

GenStat version 17 was used for QTL analysis. Three data files were used for QTL analysis e.g. phenotypic data from the field, genotypic data for framework markers, and positions of framework markers on the linkage map. QTL analysis involved four steps: (i) identification of the most appropriate model; (ii) simple interval mapping (SIM) in which main effect QTLs are calculated against the default threshold –log10(P) = 3.82 and QTL x E interaction; (iii) composite interval mapping (CIM) using SIM-derived QTLs as co-factors; and, (iv) A final scan, all candidate QTLs are compared, and the effect of each QTL is calculated.

All 140 RILs, for which both phenotyping and genotyping data were available, were used for QTL analysis. The QTL analysis was performed for yield traits (total seed yield, total biomass and 100-seed weight) and phenology traits (time to flower and length of reproductive phase). It should be noted that the linkage map was developed based on the 97 unskewed RILs as outlined previously. Comparative QTL analysis of 97 RILs and full set of 140 RILs (not presented) showed no notable differences in the resultant candidate loci thus justifying the use of all 140 RILs for QTL analysis.

## Conclusion

This study reports the first linkage map for yellow lupin, based on NGS-based genotyping methods for this species. It is also the first example of QTL analysis conducted in yellow lupin that identified QTLs for yield and phenology traits. It provides a starting point for engaging in the development of productive yellow lupin cultivars adapted to low rainfall regions of Australia and beyond.

## Additional files

Additional file 1: **Figure S1.** The diagram presenting the clustering of RILs based on their genetic distances compared to population parents. The RILs are clustering on x-axis while y-axis shows the distance measured in NTSYS software. (DOCX 140 kb)

Additional file 2: **Table S1.** Summary table of all the markers used to develop a yellow lupin map. The table comprised of marker name (SNP markers from DArT-seq appears as DArT_SNP, SNP from GBS approach appears as Scaffold and PAV markers appears as DArT-PAV), type (Framework = main skeleton markers, Redundant = exact duplicates of Framework markers and Attached = the markers which were initially not used for mapping because of high segregation distortion or missing values but later connected to closest possible location on map) and the chi-square values (expressed both in chi2 and chip values) which show the deviation from expected 1:1 ratios. (XLSX 1258 kb)

## Data Availability

The datasets used and/or analysed during the current study are available from the corresponding author on reasonable request.

## References

[1] Avise J (2012). Molecular markers, natural history and evolution: springer science & Business Edition.

[2] Schlötterer C (2004). The evolution of molecular markers—just a matter of fashion?. Nat Rev Genet.

[3] Collard B, Jahufer M, Brouwer J, Pang E (2005). An introduction to markers, quantitative trait loci (QTL) mapping and marker-assisted selection for crop improvement: the basic concepts. Euphytica.

[4] Varshney R, Bertioli D, Moretzsohn M, Vadez V, Krishnamurthy L, Aruna R, Nigam S, Moss B, Seetha K, Ravi K (2009). The first SSR-based genetic linkage map for cultivated groundnut (*Arachis hypogaea* L.). Theor Appl Genet.

[5] Varshney R, Mohan S, Gaur P, Gangarao N, Pandey M, Bohra A, Sawargaonkar S, Chitikineni A, Kimurto P, Janila P (2013). Achievements and prospects of genomics-assisted breeding in three legume crops of the semi-arid tropics. Biotechnol Adv.

[6] Kumar J, Choudhary A, Solanki R, Pratap A (2011). Towards marker-assisted selection in pulses: a review. Plant Breed.

[7] Gondo T, Sato S, Okumura K, Tabata S, Akashi R, Isobe S (2007). Quantitative trait locus analysis of multiple agronomic traits in the model legume *Lotus japonicus*. Genome.

[8] Cruz-Izquierdo S, Avila C, Satovic Z, Palomino C, Gutierrez N, Ellwood S, Phan H, Cubero J, Torres A (2012). Comparative genomics to bridge Vicia faba with model and closely-related legume species: stability of QTLs for flowering and yield-related traits. Theor Appl Genet.

[9] Herrmann D, Boller B, Studer B, Widmer F, Kölliker R (2006). QTL analysis of seed yield components in red clover (*Trifolium pratense* L.). Theor Appl Genet.

[10] Cogan N, Abberton M, Smith K, Kearney G, Marshall A, Williams A, Michaelson-Yeates T, Bowen C, Jones E, Vecchies A (2006). Individual and multi-environment combined analyses identify QTLs for morphogenetic and reproductive development traits in white clover (*Trifolium repens* L.). Theor Appl Genet.

[11] Schuster S (2007). Next-generation sequencing transforms today’s biology. Nature.

[12] Blair M, Iriarte G, Beebe S (2006). QTL analysis of yield traits in an advanced backcross population derived from a cultivated Andean × wild common bean (*Phaseolus vulgaris* L.) cross. Theor Appl Genet.

[13] Julier B, Huguet T, Chardon F, Ayadi R, Pierre J-B, Prosperi J-M, Barre P, Huyghe C (2007). Identification of quantitative trait loci influencing aerial morphogenesis in the model legume *Medicago truncatula*. Theor Appl Genet.

[14] Klein M, Grusak M (2009). Identification of nutrient and physical seed trait QTL in the model legume *Lotus japonicus*. Genome.

[15] Tar'an B, Warkentin T, Somers D, Miranda D, Vandenberg A, Blade S, Bing D (2004). Identification of quantitative trait loci for grain yield, seed protein concentration and maturity in field pea (*Pisum sativum* L.). Euphytica.

[16] Varshney R, Nayak S, May G, Jackson S (2009). Next-generation sequencing technologies and their implications for crop genetics and breeding. Trends Biotechnol.

[17] French R, Sweetingham M, Shea G (2001). A comparison of the adaptation of yellow lupin (Lupinus luteus L.) and narrow-leafed lupin (L. angustifolius L.) to acid sandplain soils in low rainfall agricultural areas of Western Australia. Aust J Agric Res.

[18] Davies C, Turner D, Dracup M (2000). Yellow lupin (Lupinus luteus) tolerates waterlogging better than narrow-leafed lupin (L. angustifolius) I. shoot and root growth in a controlled environment. Aust J Agric Res.

[19] Jones R, Latham L (1997). Natural resistance to cucumber mosaic virus in lupin species. Ann Appl Biol.

[20] French B, White P (2002). Environmental influences on lupin growth. Producing lupins; Lupin Bulletin.

[21] Glencross B, Palta J, Berger J (2008). Harvesting the benefits of lupin meals in aquaculture feeds. Lupins for health and wealth Proceedings 12th International Lupin Conference.

[22] Clements J, Chong L, Quealy J, Prilyuk L, Yang H, Francis G, Buirchell B. Interspecific hybrids between *Lupinus angustifolius* and *L. luteus*–an avenue to increase the value of narrow-leafed lupin in Australia. SABRAO J Breed Genet. 2009;41.

[23] Berger JD, Adhikari K, Wilkinson D, Buirchel B, Sweetingham M (2008). Ecogeography of the Old World lupins. 1. Ecotypic variation in yellow lupin (*Lupinus luteus* L.). Aust J Agric Res.

[24] Adhikari KN, Thomas G, Buirchell BJ, Sweetingham MW (2011). Identification of anthracnose resistance in yellow lupin (*Lupinus luteus* L.) and its incorporation into breeding lines. Plant Breed.

[25] Adhikari KN, Buirchell BJ, Sweetingham MW (2012). Length of vernalization period affects flowering time in three lupin species. Plant Breed.

[26] Parra-González L, Aravena-Abarzúa G, Navarro-Navarro C, Udall J, Maughan J, Peterson L, Salvo-Garrido H, Maureira-Butler I (2012). Yellow lupin (*Lupinus luteus* L.) transcriptome sequencing: molecular marker development and comparative studies. BMC Genomics.

[27] Foley R, Jimenez-Lopez J, Kamphuis L, Hane J, Melser S, Singh K (2015). Analysis of conglutin seed storage proteins across lupin species using transcriptomic, protein and comparative genomic approaches. BMC Plant Biol.

[28] Croxford A, Rogers T, Caligari P, Wilkinson M (2008). High-resolution melt analysis to identify and map sequence-tagged site anchor points onto linkage maps: a white lupin (*Lupinus albus*) map as an exemplar. New Phytol.

[29] O’Rourke J, Yang S, Miller S, Bucciarelli B, Liu J, Rydeen A, Bozsoki Z, Uhde-Stone C, Tu Z, Allan D (2013). An RNA-Seq transcriptome analysis of orthophosphate-deficient white lupin reveals novel insights into phosphorus acclimation in plants. Plant Physiol.

[30] Ksiazkiewicz M, Nazzicari N, Yang H, Nelson MN, Renshaw D, Rychel S, Ferrari B, Carelli M, Tomaszewska M, Stawiński S (2017). A high-density consensus linkage map of white lupin highlights synteny with narrow-leafed lupin and provides markers tagging key agronomic traits. Sci Rep.

[31] Raman R, Cowley RB, Raman H, Luckett DJ (2014). Analyses using SSR and DArT molecular markers reveal that Ethiopian accessions of white lupin (*Lupinus albus* L.) represent a unique genepool. Open J Genet.

[32] Secco D, Shou H, Whelan J, Berkowitz O (2014). RNA-seq analysis identifies an intricate regulatory network controlling cluster root development in white lupin. BMC Genomics.

[33] Hane James K., Ming Yao, Kamphuis Lars G., Nelson Matthew N., Garg Gagan, Atkins Craig A., Bayer Philipp E., Bravo Armando, Bringans Scott, Cannon Steven, Edwards David, Foley Rhonda, Gao Ling-ling, Harrison Maria J., Huang Wei, Hurgobin Bhavna, Li Sean, Liu Cheng-Wu, McGrath Annette, Morahan Grant, Murray Jeremy, Weller James, Jian Jianbo, Singh Karam B. (2016). A comprehensive draft genome sequence for lupin (Lupinus angustifolius), an emerging health food: insights into plant-microbe interactions and legume evolution. Plant Biotechnology Journal.

[34] Kamphuis L, Hane J, Nelson M, Gao L, Atkins C, Singh K (2015). Transcriptome sequencing of different narrow-leafed lupin tissue types provides a comprehensive uni-gene assembly and extensive gene-based molecular markers. Plant Biotechnol J.

[35] Zhou G, Jian J, Wang P, Li C, Tao Y, Li X, Renshaw D, Clements J, Sweetingham M, Yang H (2018). Construction of an ultra-high density consensus genetic map, and enhancement of the physical map from genome sequencing in Lupinus angustifolius. Theor Appl Genet.

[36] Mousavi-Derazmahalleh M, Bayer PE, Nevado B, Hurgobin B, Filatov D, Kilian A, Kamphuis LG, Singh KB, Berger JD, Hane JK (2018). Exploring the genetic and adaptive diversity of a pan-Mediterranean crop wild relative: narrow-leafed lupin. Theor Appl Genet.

[37] Taylor Candy M., Kamphuis Lars G., Zhang Weilu, Garg Gagan, Berger Jens D., Mousavi-Derazmahalleh Mahsa, Bayer Philipp E., Edwards David, Singh Karam B., Cowling Wallace A., Nelson Matthew N. (2018). INDEL variation in the regulatory region of the major flowering time gene LanFTc1 is associated with vernalization response and flowering time in narrow-leafed lupin (Lupinus angustifolius L.). Plant, Cell & Environment.

[38] Mousavi-Derazmahalleh M, Nevado B, Bayer PE, Filatov DA, Hane JK, Edwards D, Erskine W, Nelson MN (2018). The western Mediterranean region provided the founder population of domesticated narrow-leafed lupin. Theor Appl Genet.

[39] Berger JD, Buirchell B, Luckett D, Palta J, Ludwig C, Liu D (2012). How has narrow-leafed lupin changed in its 1st 40 years as an industrial, broad-acre crop? A GxE-based characterization of yield-related traits in Australian cultivars. Field Crop Res.

[40] Berger JD, John C, Nelson M, Kamphuis L, Singh K, Buirchell B (2013). The essential role of genetic resources in narrow-leafed lupin improvement. Crop Pasture Sci.

[41] Gao L-L, Hane J, Kamphuis L, Foley R, Shi B, Atkins C, Singh K (2011). Development of genomic resources for the narrow-leafed lupin *(Lupinus angustifolius*): construction of a bacterial artificial chromosome (BAC) library and BAC-end sequencing. BMC Genomics.

[42] Vadez V, Berger J, Warkentin T, Asseng S, Ratnakumar P, Rao K, Gaur P, Munier-Jolain N, Larmure A, Voisin A-S (2012). Adaptation of grain legumes to climate change: a review. Agron Sustain Dev.

[43] Nielsen R, Paul J, Albrechtsen A, Song Y (2011). Genotype and SNP calling from next-generation sequencing data. Nat Rev Genet.

[44] Davey J, Hohenlohe P, Etter P, Boone J, Catchen J, Blaxter M (2011). Genome-wide genetic marker discovery and genotyping using next-generation sequencing. Nat Rev Genet.

[45] Mester David, Ronin Yefim, Schnable Patrick, Aluru Srinivas, Korol Abraham (2015). Fast and Accurate Construction of Ultra-Dense Consensus Genetic Maps Using Evolution Strategy Optimization. PLOS ONE.

[46] Poland J, Brown P, Sorrells M, Jannink J-L (2012). Development of high-density genetic maps for barley and wheat using a novel two-enzyme genotyping-by-sequencing approach. PLoS One.

[47] Rutkowski L, Gonzales E, von Davier M, Zhou Y (2014). Assessment design for international large-scale assessments. Handbook of international large-scale assessment: Background, technical issues, and methods of data analysis.

[48] Schmitt P, Mandel J, Guedj M (2015). A comparison of six methods for missing data imputation. J Biom Biostat.

[49] Gardner K, Brown P, Cooke T, Cann S, Costa F, Bustamante C, Velasco R, Troggio M, Myles S (2014). Fast and cost-effective genetic mapping in apple using next-generation sequencing. G3.

[50] Hamblin M, Rabbi I (2014). The effects of restriction-enzyme choice on properties of genotyping-by-sequencing libraries: a study in cassava. Crop Sci.

[51] Sonah H, Bastien M, Iquira E, Tardivel A, Légaré G, Boyle B, Normandeau É, Laroche J, Larose S, Jean M (2013). An improved genotyping by sequencing (GBS) approach offering increased versatility and efficiency of SNP discovery and genotyping. PLoS One.

[52] Nelson MN, Moolhuijzen P, Boersma J, Chudy M, Lesniewska K, Bellgard M, Oliver R, Swiecicki W, Wolko B, Cowling W (2010). Aligning a new reference genetic map of *Lupinus angustifolius* with the genome sequence of the model legume, Lotus japonicus. DNA Res.

[53] Fu Y-B (2014). Genetic diversity analysis of highly incomplete SNP genotype data with imputations: an empirical assessment. G3.

[54] Poland J, Rife T (2012). Genotyping-by-sequencing for plant breeding and genetics. Plant Genome.

[55] Nelson MN, Phan H, Ellwood S, Moolhuijzen P, Hane J, Williams A, Clare E, Fosu-Nyarko J, Scobie M, Cakir M (2006). The first gene-based map of *Lupinus angustifolius* L.-location of domestication genes and conserved synteny with *Medicago truncatula*. Theor Appl Genet.

[56] Eujayl I, Baum M, Erskine W, Pehu E, Muehlbauer F (1997). The use of RAPD markers for lentil genetic mapping and the evaluation of distorted F2 segregation. Euphytica.

[57] Zamir D, Tadmor Y (1986). Unequal segregation of nuclear genes in plants. Bot Gaz.

[58] He J, Zhao X, Laroche A, Lu Z-X, Liu H, Li Z. Genotyping-by-sequencing (GBS), an ultimate marker-assisted selection (MAS) tool to accelerate plant breeding. Front Plant Sci. 2014;5. 10.3389/fpls.2014.00484 PMC4179701 25324846

[59] Rafalski A (2002). Applications of single nucleotide polymorphisms in crop genetics. Curr Opin Biotechnol.

[60] Berger JD, Ludwig C (2014). Contrasting adaptive strategies to terminal drought-stress gradients in Mediterranean legumes: phenology, productivity, and water relations in wild and domesticated *Lupinus luteus* L. J Exp Bot.

[61] Jinks J, Pooni H (1976). Predicting the properties of recombinant inbred lines derived by single seed descent. Heredity.

[62] Joshi N, Fass J (2011). Sickle-a windowed adaptive trimming tool for FASTQ files using quality.

[63] Wu T, Nacu S (2010). Fast and SNP-tolerant detection of complex variants and splicing in short reads. Bioinformatics.

[64] Luo R, Liu B, Xie Y, Li Z, Huang W, Yuan J, He G, Chen Y, Pan Q, Liu Y (2012). SOAPdenovo2: an empirically improved memory-efficient short-read de novo assembler. Gigascience.

[65] Li H, Handsaker B, Wysoker A, Fennell T, Ruan J, Homer N, Marth G, Abecasis G, Durbin R (2009). The sequence alignment/map format and SAMtools. Bioinformatics.

[66] Page J, Liechty Z, Huynh M, Udall J (2014). BamBam: genome sequence analysis tools for biologists. BMC Res Notes.

[67] Doyle J, Doyle J (1987). Genomic plant DNA preparation from fresh tissue-CTAB method. Phytochem Bull.

[68] Mester D, Ronin Y, Hu Y, Peng J, Nevo E, Korol A (2003). Efficient multipoint mapping: making use of dominant repulsion-phase markers. Theor Appl Genet.

[69] Rohlf F (1992). NTSYS-pc: numerical taxonomy and multivariate analysis system: applied biostatistics Inc.

[70] Clarke K, Gorley R (2006). PRIMER v6: user manual/tutorial.

[71] Voorrips R (2002). MapChart: software for the graphical presentation of linkage maps and QTLs. J Hered.

[72] Russell C, Fillery I (1996). Estimates of lupin below-ground biomass nitrogen, dry matter, and nitrogen turnover to wheat. Crop Pasture Sci.

[73] Coombes N. DiGGer design search tool in R: New South Wales Department of Primary Industry; 2009. Available at http://nswdpibiom.org/austatgen/software/ [Verified 29 Aug 2017]

[74] Falconer D (1960). Introduction to quantitative genetics: DS Falconer.

[75] Snedecor G, Cochran W (1967). Statistical Methods.

