# Supplementary material for: The first genetic map for yellow lupin enables genetic dissection of adaptation traits in an orphan grain legume crop
Source: BMC Genet. 2019 Aug 14;20:68. doi: 10.1186/s12863-019-0767-3 (PMC6694670; doi:10.1186/s12863-019-0767-3)
Supplement: Supplementary file 1 — Figure S1. The diagram presenting the clustering of RILs based on their genetic distances compared to population parents. The RILs are clustering on x-axis while y-axis shows the distance measured in NTSYS software. (DOCX 140 kb) [file 12863_2019_767_MOESM1_ESM.docx]

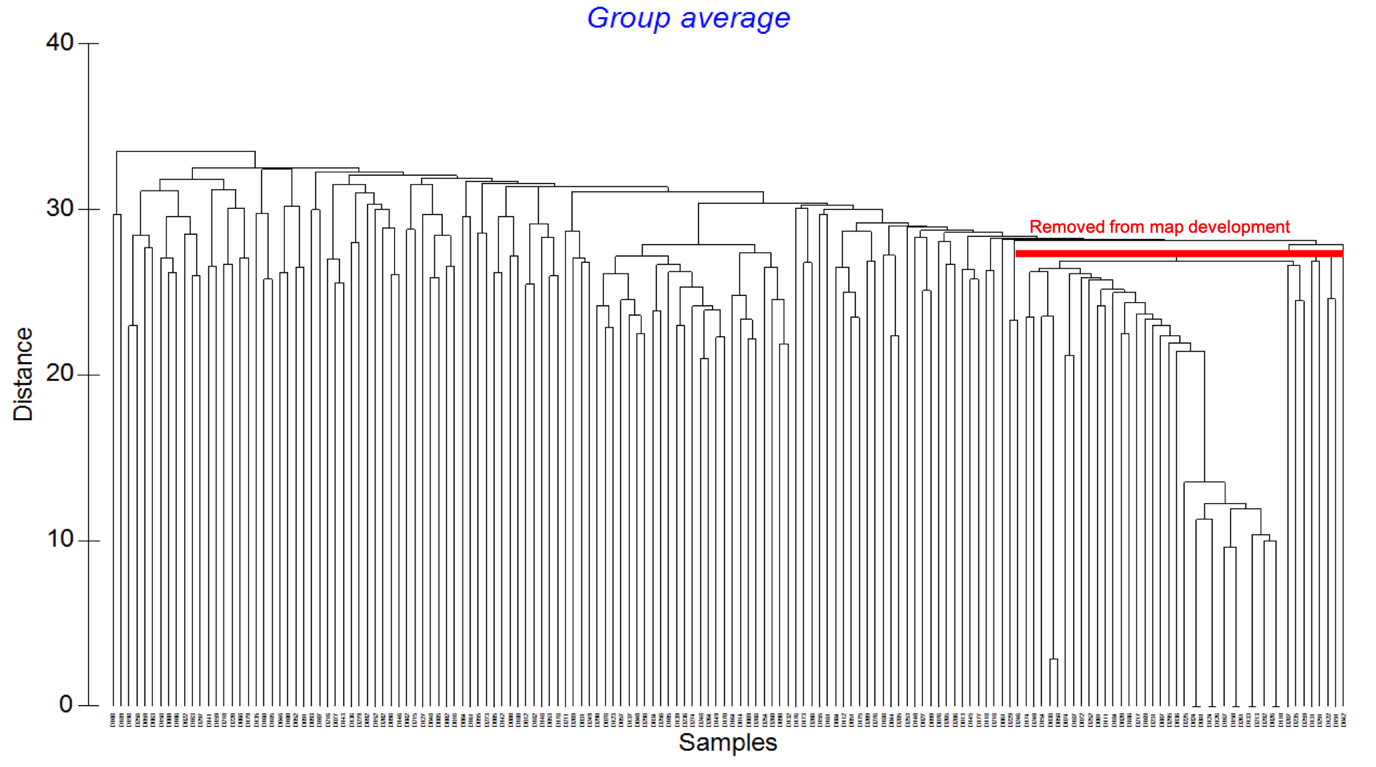


**Supplementary Figure 1:** The diagram presenting the clustering of RILs based on their genetic distances compared to population parents. The RILs are clustering on x-axis while y-axis shows the distance measured in NTSYS software
